# Supplementary material for: Enhanced Catalytic Activity of TEMPO-Mediated Aerobic Oxidation of Alcohols via Redox-Active Metal–Organic Framework Nodes
Source: Molecules. 2023 Jan 6;28(2):593. doi: 10.3390/molecules28020593 (PMC9865133; doi:10.3390/molecules28020593)
Supplement: Supplementary file 1 [file molecules-28-00593-s001.zip › molecules-2103424-supplementary.pdf]

Supplementary Materials

Enhanced Catalytic Activity of TEMPO-Mediated Aerobic Oxidation of Alcohols via Redox-Active Metal–Organic Framework Nodes

Bing Wang [1,†], Junjie Zhang [1,†], Yun Xue [1], Yuliang Chong [1], Dongdong Zhao [1], Hu Cheng [1], Liangliang Tian [2] and Jinliang Zhuang [1,*]

1. General Methods and Materials

All the chemicals were obtained from commercial sources, unless otherwise noted, and used without further purification. All of the reactions and manipulations were carried out under nitrogen with the use of standard inert atmosphere. Column chromatography was performed with 200-300 mesh neutral silica gels. The sorption isotherm measurements were performed on an ASAP (Accelerated Surface Area and Porosimetry) 2020 System. The Brunauer-Emmett-Teller (BET) method was utilized to calculate the specific surface areas. The pore volume and pore size distribution were derived from the sorption curve by using the non-local density functional theory (NLDFT) model. Before gas adsorption measurement, sample was dried again using the "outgas" function of the surface area analyzer for 5 h at 313 K. Powder X-ray diffractions (PXRD) were recorded on a Rigaku Ultima IV diffractometer using Cu Kα 1 (1.5418 Å) radiation, from 3-50°. FT-IR spectrum were collected with a NICOLET 6700 Fourier Transform Infrared Spectrometer at a resolution 4 cm-1. SEM images were recorded using Hitachi S-4800. GC analysis was measured on GC-9160. General GC conditions for: Restek column, 30 m × 0.32 mm × 0.5mm; FID detector; carrier gas: N_2; area normalization. Column Conditions for a benzyl alcohol, 4-methoxybenzyl alcohol, 4-tert-butylbenzyl alcohol, 4-fluorobenzyl alcohol, benzhydrol, α-methylbenzyl alcohol, 2-thiophenemethanol, furfury alcohol, 1-indanol and their corresponding carbonyl compounds: column temperature: 80 °C for 5 min, rising to 250 °C at

a rate of 20 °C/min.

2. Synthesis of functional nitroxide radicals

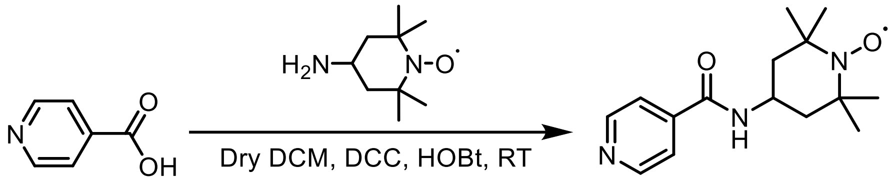

Scheme S1. Synthesis of TEMPO-IsoNTA.

TEMPO-IsoNTA was synthesized based on the method in the previous article with slight modification [1]. DCC (742.2 mg, 3.6 mmol), HOBt (486.5 mg, 3.6 mmol) were successively added to a solution of isonicotinic acid (369.3 mg, 3.0 mmol) in anhydrous CH_2Cl_2 (15 ml) under 0 °C (ice bath), and stirred for 30 min under nitrogen atmosphere. Then 4-amino-TEMPO (513.8 mg, 3.0 mmol) in anhydrous CH_2Cl_2 (7.5 ml) was added dropwise to above solution. The mixture was warmed to room temperature and further stirred 12 h under nitrogen atmosphere. The reaction mixture was filtered through a Celite pad and washed with CH_2Cl_2. The solution was evaporated to get the crude product in the form of thick oil, which was purified through a silica gel column (CH_2Cl_2 : MeOH = 50:1) to collect pure product as the orange solid (623 mg, 75%). ^1H NMR (DMSO-d_6 with phenylhydrazien, 500MHz): δ (ppm) = 8.70 (d, 2H), 7.72 (d, 2H), 7.71 (s, 1H), 4.02 (g, 1H), 1.74 (d, 2H), 1.50 (d, 2H), 1.10 (d, 12H). ^{13}C NMR (DMSO-d6 with phenylhydrazien, 500MHz): δc (ppm) = 164.47, 150.58, 128.77, 121.71, 58.40, 45.04, 33.13, 20.10. MALDI-TOF-MS: m/z Calcd m/z 277.17 [M+H]+ Found m/z 276.17.

3. Characterizations of TEMPO-IsoNTA and MOFs

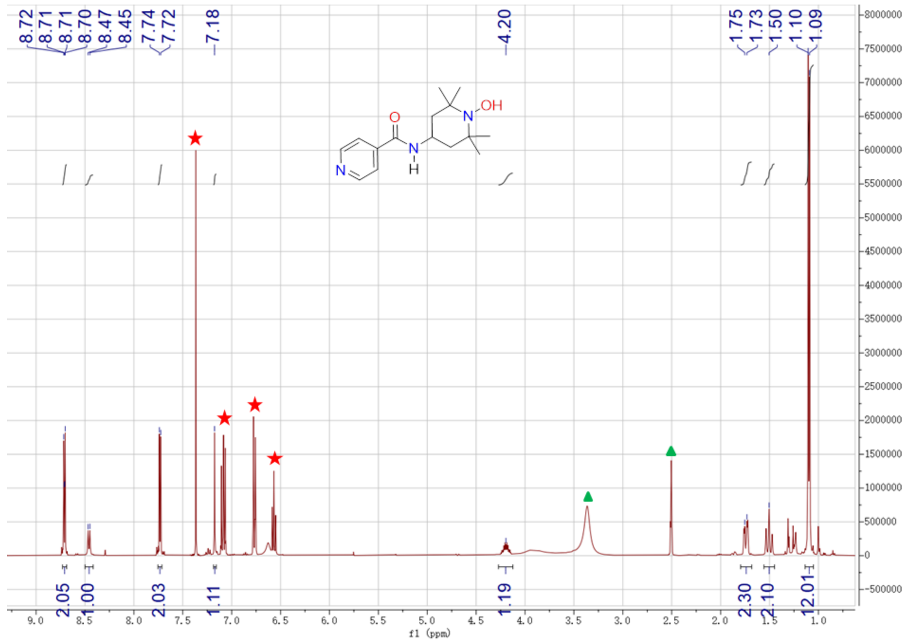

Figure S1: ^{1}H NMR spectrum of TEMPO-IsoNTA in a DMSO-d6 solution with addition of phenylhydrazine (5 μL). δ (ppm) = 8.70 (d, 2H), 7.72 (d, 2H), 7.17 (s, 1H), 4.02 (g, 1H), 1.74 (d, 2H), 1.50 (d, 2H), 1.10 (d, 12H). The stars show the HNMR peaks of phenylhydrazine.

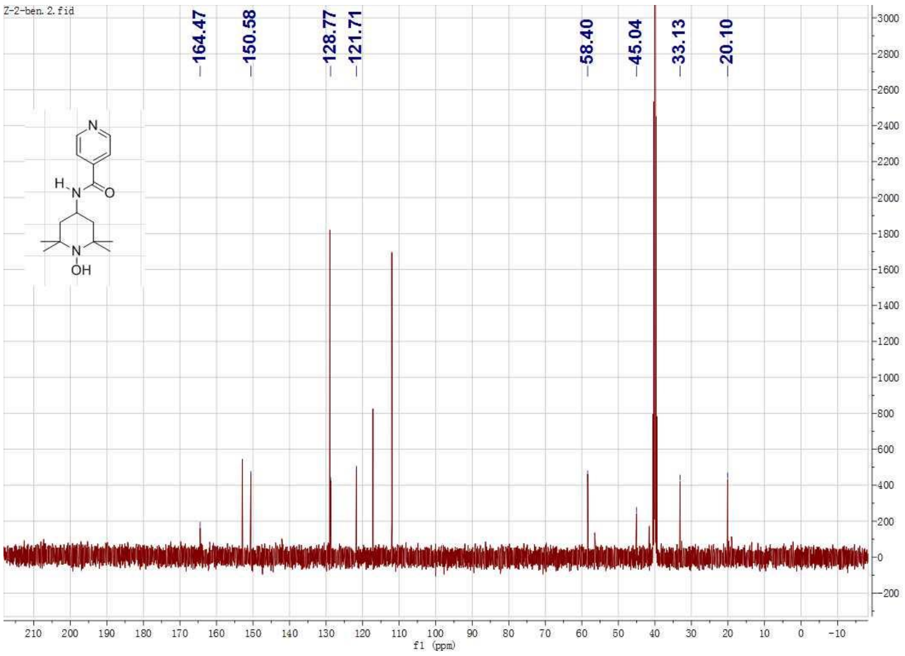

Figure S2: ^{13}C NMR spectrum of TEMPO-IsoNTA in a DMSO-d6 solution with addition of phenylhydrazine (5 μL). δc (ppm) = 164.47, 150.58, 128.77, 121.71, 58.40, 45.04, 33.13, 20.10.

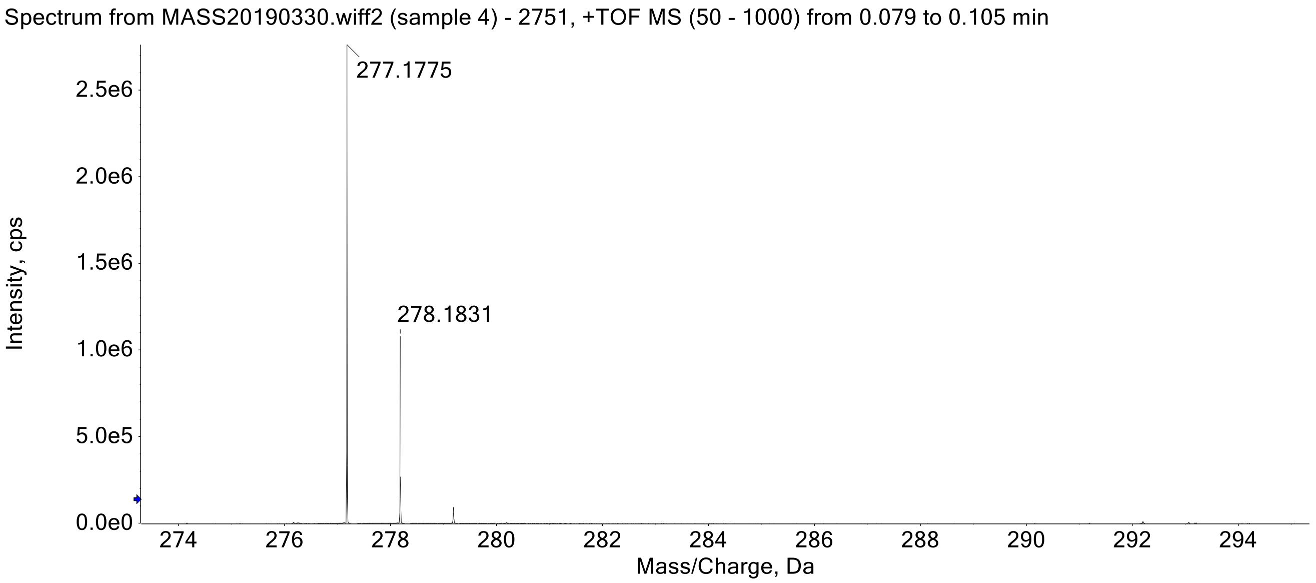

Figure S3: MS spectrum of the TEMPO-IsoNTA, MALDI-TOF-MS: m/z: 277.1775, [M+H]= 277.17, Calculated value: 276.17.

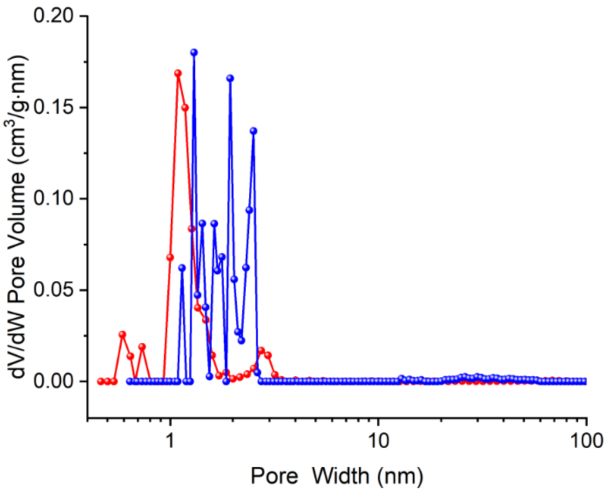

Figure S4: Pore size distribution of MIL-101(Fe) (red curve) and MIL-101(Cr) (blue curve) calculated by NLDFT.

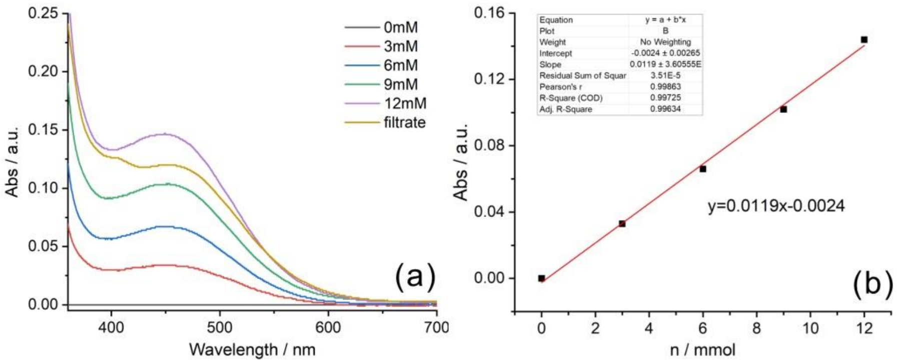

Figure S5: (a) UV-Vis spectrum (filtrate: the clear solution after adsorbing from MIL-101(Fe)) and (b) standard curve of TEMPO-IsoNTA.

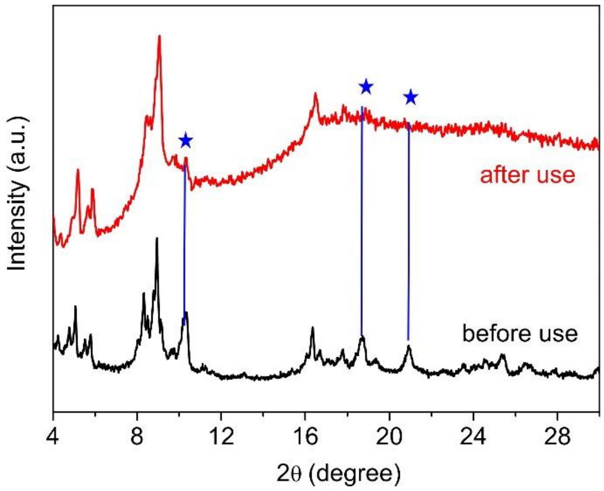

Figure S6: XRD pattern of MIL-101(Fe) before (black) and after use (red).

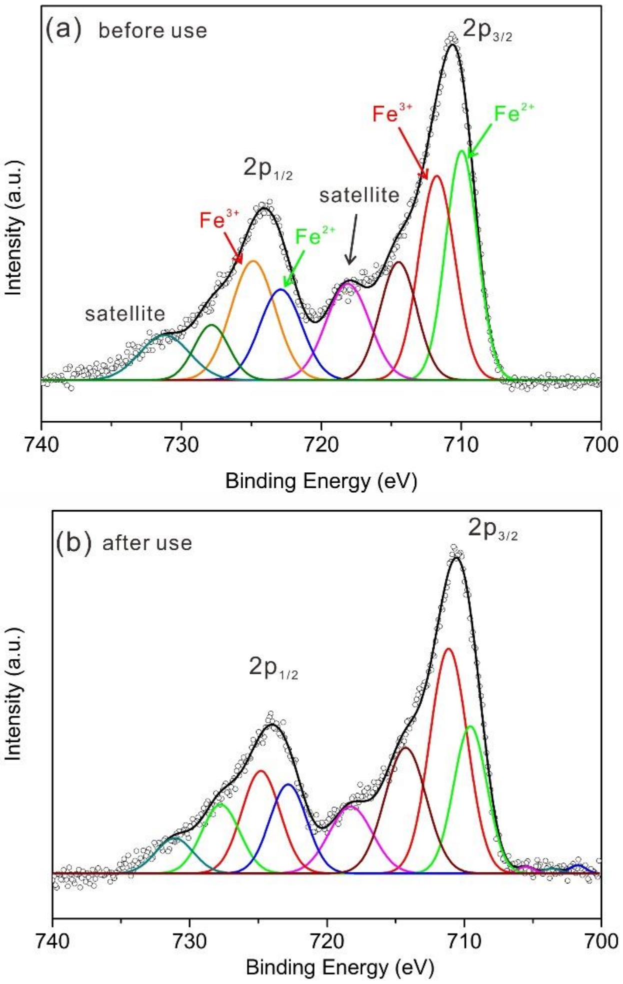

Figure S7: High-resolution Fe 2p XPS spectra of MIL-101(Fe) before (a) and after use (b).

4. Catalysis studies

Typical procedure for aerobic oxidation of alcohols: Added benzyl alcohol (0.3 mmol), MIL-101(Fe/Cr) (0.015 mmol), TEMPO-IsoNTA (0.015 mmol),

$PhCF_3$ (1.5 ml, saturated with O_2), TBN (0.06 mmol) to a 10 mL screw capped vial, then the vial was sealed and placed in a temperature regulated oven (80 °C) for a period of time. After cooling to room temperature, p-nitrobenzene (internal standard, 0.3 mmol) was added and the catalyst was removed by centrifugation. The clear solution was analyzed by GC without further purification. For the recycling experiments, benzyl alcohol was used as the substrate. The catalysts were collected by centrifugation, washed twice with ethanol and dried under vacuum.

5. References

1. Banerjee, M.; Das, S.; Yoon, M.; Choi, H.J.; Hyun, M.H.; Park, S.M.; Seo, G.; Kim, K. Postsynthetic Modification Switches an Achiral Framework to Catalytically Active Homochiral Metal-Organic Porous Materials. *J. Am. Chem. Soc.* **2009**, *131*, 7524–7525.
